# Crystal structure of (*E*)-*N*′-(3,4-di­fluoro­benzyl­idene)-4-methyl­benzene­sulfono­hydrazide

**DOI:** 10.1107/S2056989015016205

**Published:** 2015-09-17

**Authors:** Yeming Wang, Hong Yan

**Affiliations:** a College of Life Science and Bio-engineering, Beijing University of Technology, No. 100 Pingleyuan, Chaoyang District, Beijing 100124, People’s Republic of China

**Keywords:** crystal structure, sulfonyl­hydrazone, hydrogen bonding, biological activity

## Abstract

In the title compound, C_14_H_12_F_2_N_2_O_2_S, the dihedral angle between the aromatic rings is 70.23 (8)° and the S—N—N=C torsion angle is 172.11 (11)°. In the crystal, N—H⋯O hydrogen bonds link the mol­ecules into [100] *C*(4) chains, with adjacent mol­ecules in the chain related by translational symmetry. The chains are linked by weak C—H⋯F and C—H⋯O inter­actions, thereby forming a three-dimensional network.

## Related literature   

For the biological activities of sulfonamides and sulfonyl­hydrazones, see: El-Sayed *et al.* (2011 ▸); de Oliveira *et al.* (2011 ▸); Zhao *et al.* (2011 ▸).
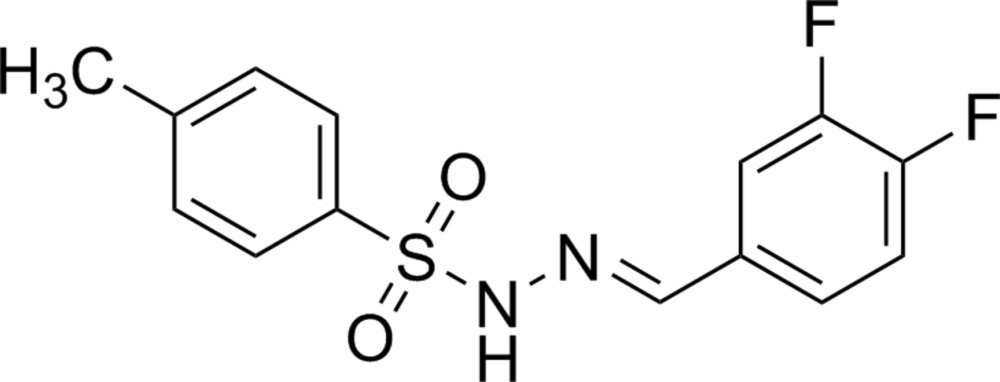


## Experimental   

### Crystal data  

C_14_H_12_F_2_N_2_O_2_S
*M* *_r_* = 310.32
Monoclinic, 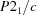

*a* = 5.3161 (11) Å
*b* = 15.388 (3) Å
*c* = 17.293 (4) Å
β = 97.44 (3)°
*V* = 1402.8 (5) Å^3^
*Z* = 4
Mo *K*α radiation
μ = 0.26 mm^−1^
*T* = 173 K
0.20 × 0.18 × 0.12 mm

### Data collection  

Rigaku Saturn diffractometer
Absorption correction: multi-scan (*CrystalClear*; Rigaku, 2009 ▸) *T*_min_ = 0.950, *T*_max_ = 0.970
14131 measured reflections
3328 independent reflections
2729 reflections with *I* > 2σ(*I*)
*R*_int_ = 0.047

### Refinement  

*R*[*F* ^2^ > 2σ(*F* ^2^)] = 0.039
*wR*(*F* ^2^) = 0.116
*S* = 1.04
3328 reflections
196 parameters
1 restraint
H atoms treated by a mixture of independent and constrained refinement
Δρ_max_ = 0.27 e Å^−3^
Δρ_min_ = −0.39 e Å^−3^

### 

Data collection: *CrystalClear* (Rigaku, 2009 ▸); cell refinement: *CrystalClear*; data reduction: *CrystalClear*; program(s) used to solve structure: *SHELXS97* (Sheldrick, 2008 ▸); program(s) used to refine structure: *SHELXL97* (Sheldrick, 2008 ▸); molecular graphics: *SHELXTL* (Sheldrick, 2008 ▸); software used to prepare material for publication: *SHELXTL*.

## Supplementary Material

Crystal structure: contains datablock(s) I, New_Global_Publ_Block. DOI: 10.1107/S2056989015016205/hb7491sup1.cif

Structure factors: contains datablock(s) I. DOI: 10.1107/S2056989015016205/hb7491Isup2.hkl

Supporting information file. DOI: 10.1107/S2056989015016205/hb7491Isup3.cml

**Fig. 1.**
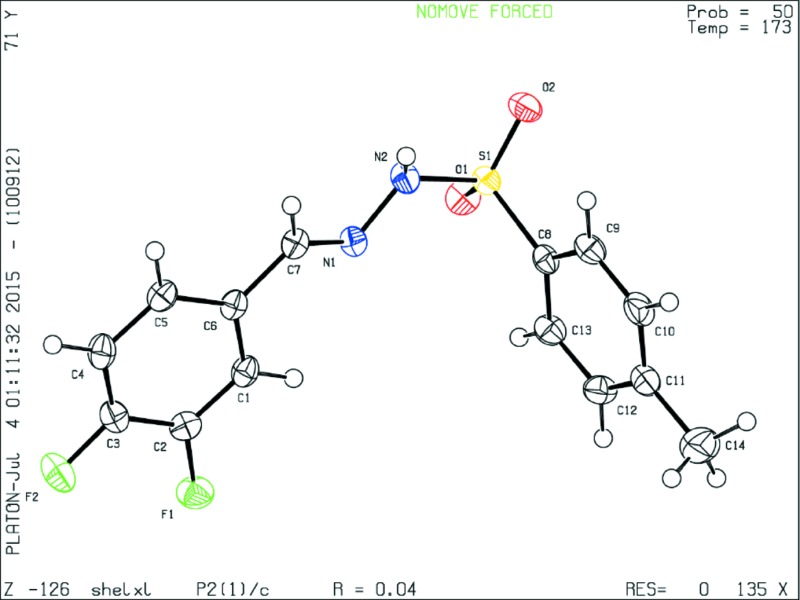
Displacement ellipsoid plot (50% probability level) of the title compound.

. DOI: 10.1107/S2056989015016205/hb7491fig1.tif
Displacement ellipsoid plot (50% probability level) of the title compound.

CCDC reference: 1040450

Additional supporting information:  crystallographic information; 3D view; checkCIF report

## Figures and Tables

**Table 1:** Hydrogen-bond geometry (, )

*D*H*A*	*D*H	H*A*	*D* *A*	*D*H*A*
N2H2O1^i^	0.89(1)	2.09(1)	2.9747(18)	174(2)
C1H1F1^ii^	0.95	2.47	3.345(2)	153
C4H4O2^iii^	0.95	2.57	3.473(2)	159

Symmetry codes: (i) 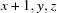; (ii) 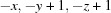; (iii) 
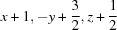
.

## supplementary crystallographic information

### S1.1. Synthesis and crystallization

p-Tosyl­hydrazine (0.372 g, 2 mmol) was added to a 50 ml refluxing ethano­lic solution of 3,4-di­fluoro­benzaldehyde (0.284 g, 2 mmol). The mixture was stirred for 3 h. After cooling, the colorless crystalline solid was isolated by filtration, washed with cold ethanol, and recrystallized from ethanol as colourless prisms.

### Crystal data

**Table:**

C_14_H_12_F_2_N_2_O_2_S	*D*_x_ = 1.469 Mg m^−^^3^
*M**_r_* = 310.32	Melting point: 426 K
Monoclinic, *P*2_1_/*c*	Mo *K*α radiation, λ = 0.71073 Å
*a* = 5.3161 (11) Å	Cell parameters from 3967 reflections
*b* = 15.388 (3) Å	θ = 1.8–27.9°
*c* = 17.293 (4) Å	µ = 0.26 mm^−^^1^
β = 97.44 (3)°	*T* = 173 K
*V* = 1402.8 (5) Å^3^	Prism, colourless
*Z* = 4	0.20 × 0.18 × 0.12 mm
*F*(000) = 640	

### Data collection

**Table:**

Rigaku Saturn diffractometer	3328 independent reflections
Radiation source: rotating anode	2729 reflections with *I* > 2σ(*I*)
Confocal monochromator	*R*_int_ = 0.047
ω scans	θ_max_ = 27.9°, θ_min_ = 1.8°
Absorption correction: multi-scan (*CrystalClear*; Rigaku, 2009)	*h* = −6→6
*T*_min_ = 0.950, *T*_max_ = 0.970	*k* = −20→19
14131 measured reflections	*l* = −22→22

### Refinement

**Table:**

Refinement on *F*^2^	Secondary atom site location: difference Fourier map
Least-squares matrix: full	Hydrogen site location: inferred from neighbouring sites
*R*[*F*^2^ > 2σ(*F*^2^)] = 0.039	H atoms treated by a mixture of independent and constrained refinement
*wR*(*F*^2^) = 0.116	*w* = 1/[σ^2^(*F*_o_^2^) + (0.0715*P*)^2^ + 0.0398*P*] where *P* = (*F*_o_^2^ + 2*F*_c_^2^)/3
*S* = 1.04	(Δ/σ)_max_ = 0.001
3328 reflections	Δρ_max_ = 0.27 e Å^−^^3^
196 parameters	Δρ_min_ = −0.39 e Å^−^^3^
1 restraint	Extinction correction: *SHELXL97* (Sheldrick, 2008), Fc^*^=kFc[1+0.001xFc^2^λ^3^/sin(2θ)]^-1/4^
Primary atom site location: structure-invariant direct methods	Extinction coefficient: 0.057 (4)

### Special details

**Table:**

Geometry. All e.s.d.'s (except the e.s.d. in the dihedral angle between two l.s. planes) are estimated using the full covariance matrix. The cell e.s.d.'s are taken into account individually in the estimation of e.s.d.'s in distances, angles and torsion angles; correlations between e.s.d.'s in cell parameters are only used when they are defined by crystal symmetry. An approximate (isotropic) treatment of cell e.s.d.'s is used for estimating e.s.d.'s involving l.s. planes.
Refinement. Refinement of *F*^2^ against ALL reflections. The weighted *R*-factor *wR* and goodness of fit *S* are based on *F*^2^, conventional *R*-factors *R* are based on *F*, with *F* set to zero for negative *F*^2^. The threshold expression of *F*^2^ > σ(*F*^2^) is used only for calculating *R*-factors(gt) *etc*. and is not relevant to the choice of reflections for refinement. *R*-factors based on *F*^2^ are statistically about twice as large as those based on *F*, and *R*- factors based on ALL data will be even larger.

### Fractional atomic coordinates and isotropic or equivalent isotropic displacement parameters (Å^2^)

**Table:**

	*x*	*y*	*z*	*U*_iso_*/*U*_eq_	
S1	0.17639 (7)	0.83591 (2)	0.34262 (2)	0.02258 (15)	
F1	0.15725 (18)	0.45775 (6)	0.58632 (6)	0.0354 (3)	
F2	0.52921 (19)	0.43477 (7)	0.70237 (6)	0.0403 (3)	
O1	−0.0478 (2)	0.83384 (7)	0.38043 (6)	0.0300 (3)	
O2	0.2561 (2)	0.91533 (7)	0.31005 (6)	0.0308 (3)	
N1	0.3852 (2)	0.73655 (8)	0.45229 (7)	0.0243 (3)	
N2	0.4159 (2)	0.81054 (9)	0.40836 (7)	0.0254 (3)	
C1	0.3568 (3)	0.58214 (10)	0.54189 (9)	0.0245 (3)	
H1	0.2203	0.5908	0.5014	0.029*	
C2	0.3516 (3)	0.51491 (10)	0.59364 (9)	0.0245 (3)	
C3	0.5465 (3)	0.50210 (10)	0.65353 (9)	0.0268 (3)	
C4	0.7535 (3)	0.55625 (10)	0.66269 (9)	0.0286 (3)	
H4	0.8868	0.5476	0.7042	0.034*	
C5	0.7641 (3)	0.62404 (10)	0.60986 (9)	0.0257 (3)	
H5	0.9075	0.6614	0.6148	0.031*	
C6	0.5666 (3)	0.63768 (9)	0.54969 (8)	0.0218 (3)	
C7	0.5820 (3)	0.71137 (10)	0.49678 (8)	0.0233 (3)	
H7	0.7391	0.7405	0.4956	0.028*	
C8	0.1461 (3)	0.75599 (9)	0.26968 (8)	0.0224 (3)	
C9	0.3114 (3)	0.75831 (10)	0.21365 (9)	0.0274 (3)	
H9	0.4410	0.8011	0.2162	0.033*	
C10	0.2849 (3)	0.69783 (11)	0.15446 (9)	0.0323 (4)	
H10	0.3983	0.6992	0.1163	0.039*	
C11	0.0951 (4)	0.63458 (10)	0.14931 (10)	0.0331 (4)	
C12	−0.0658 (3)	0.63306 (11)	0.20640 (10)	0.0315 (4)	
H12	−0.1949	0.5901	0.2041	0.038*	
C13	−0.0418 (3)	0.69311 (10)	0.26667 (9)	0.0270 (3)	
H13	−0.1528	0.6912	0.3055	0.032*	
C14	0.0698 (5)	0.56939 (13)	0.08375 (12)	0.0565 (6)	
H14A	0.1822	0.5199	0.0982	0.085*	
H14B	0.1170	0.5968	0.0365	0.085*	
H14C	−0.1062	0.5491	0.0739	0.085*	
H2	0.573 (2)	0.8215 (12)	0.3993 (12)	0.043 (6)*	

### Atomic displacement parameters (Å^2^)

**Table:**

	*U*^11^	*U*^22^	*U*^33^	*U*^12^	*U*^13^	*U*^23^
S1	0.0188 (2)	0.0234 (2)	0.0255 (2)	0.00121 (13)	0.00278 (14)	0.00296 (13)
F1	0.0271 (5)	0.0342 (6)	0.0442 (6)	−0.0066 (4)	0.0022 (4)	0.0015 (4)
F2	0.0390 (6)	0.0407 (6)	0.0410 (6)	0.0035 (4)	0.0041 (5)	0.0171 (4)
O1	0.0187 (6)	0.0373 (7)	0.0352 (6)	0.0019 (4)	0.0076 (5)	−0.0028 (5)
O2	0.0332 (7)	0.0237 (6)	0.0354 (6)	−0.0007 (5)	0.0037 (5)	0.0055 (4)
N1	0.0255 (7)	0.0257 (6)	0.0219 (6)	0.0010 (5)	0.0036 (5)	0.0019 (5)
N2	0.0179 (7)	0.0307 (7)	0.0274 (7)	−0.0027 (5)	0.0021 (5)	0.0064 (5)
C1	0.0213 (8)	0.0289 (8)	0.0226 (7)	0.0036 (6)	−0.0003 (6)	−0.0028 (6)
C2	0.0194 (8)	0.0261 (8)	0.0288 (8)	−0.0010 (6)	0.0054 (6)	−0.0034 (6)
C3	0.0288 (8)	0.0275 (8)	0.0250 (7)	0.0057 (6)	0.0068 (6)	0.0033 (6)
C4	0.0246 (8)	0.0355 (9)	0.0247 (7)	0.0060 (6)	−0.0013 (6)	0.0008 (6)
C5	0.0186 (7)	0.0305 (8)	0.0272 (7)	0.0005 (6)	0.0005 (6)	−0.0031 (6)
C6	0.0198 (8)	0.0255 (7)	0.0203 (7)	0.0041 (6)	0.0030 (6)	−0.0032 (5)
C7	0.0202 (8)	0.0283 (8)	0.0217 (7)	−0.0001 (6)	0.0036 (6)	−0.0022 (6)
C8	0.0204 (8)	0.0237 (7)	0.0228 (7)	0.0030 (6)	0.0010 (6)	0.0053 (5)
C9	0.0262 (9)	0.0268 (8)	0.0303 (8)	−0.0011 (6)	0.0075 (6)	0.0064 (6)
C10	0.0424 (10)	0.0314 (9)	0.0253 (8)	0.0008 (7)	0.0126 (7)	0.0057 (6)
C11	0.0471 (11)	0.0255 (8)	0.0263 (8)	0.0016 (7)	0.0036 (7)	0.0040 (6)
C12	0.0333 (9)	0.0257 (8)	0.0350 (9)	−0.0049 (7)	0.0028 (7)	0.0017 (6)
C13	0.0228 (8)	0.0281 (8)	0.0310 (8)	−0.0020 (6)	0.0067 (6)	0.0041 (6)
C14	0.0924 (18)	0.0407 (11)	0.0384 (11)	−0.0107 (11)	0.0163 (11)	−0.0076 (8)

### Geometric parameters (Å, º)

**Table:**

S1—O1	1.4319 (12)	C5—H5	0.9500
S1—O2	1.4324 (11)	C6—C7	1.466 (2)
S1—N2	1.6404 (14)	C7—H7	0.9500
S1—C8	1.7542 (15)	C8—C13	1.387 (2)
F1—C2	1.3502 (18)	C8—C9	1.390 (2)
F2—C3	1.3473 (18)	C9—C10	1.377 (2)
N1—C7	1.2762 (19)	C9—H9	0.9500
N1—N2	1.3901 (17)	C10—C11	1.396 (3)
N2—H2	0.888 (9)	C10—H10	0.9500
C1—C2	1.371 (2)	C11—C12	1.388 (2)
C1—C6	1.398 (2)	C11—C14	1.507 (2)
C1—H1	0.9500	C12—C13	1.386 (2)
C2—C3	1.381 (2)	C12—H12	0.9500
C3—C4	1.373 (2)	C13—H13	0.9500
C4—C5	1.393 (2)	C14—H14A	0.9800
C4—H4	0.9500	C14—H14B	0.9800
C5—C6	1.395 (2)	C14—H14C	0.9800
			
O1—S1—O2	120.29 (6)	N1—C7—C6	120.22 (13)
O1—S1—N2	107.18 (7)	N1—C7—H7	119.9
O2—S1—N2	103.48 (7)	C6—C7—H7	119.9
O1—S1—C8	107.96 (7)	C13—C8—C9	120.60 (14)
O2—S1—C8	108.65 (7)	C13—C8—S1	120.99 (12)
N2—S1—C8	108.80 (7)	C9—C8—S1	118.40 (11)
C7—N1—N2	115.66 (12)	C10—C9—C8	119.19 (14)
N1—N2—S1	115.99 (10)	C10—C9—H9	120.4
N1—N2—H2	115.6 (13)	C8—C9—H9	120.4
S1—N2—H2	119.9 (13)	C9—C10—C11	121.46 (15)
C2—C1—C6	118.81 (14)	C9—C10—H10	119.3
C2—C1—H1	120.6	C11—C10—H10	119.3
C6—C1—H1	120.6	C12—C11—C10	118.25 (15)
F1—C2—C1	120.70 (14)	C12—C11—C14	121.39 (16)
F1—C2—C3	117.91 (14)	C10—C11—C14	120.36 (16)
C1—C2—C3	121.38 (14)	C13—C12—C11	121.22 (15)
F2—C3—C4	120.86 (14)	C13—C12—H12	119.4
F2—C3—C2	118.32 (14)	C11—C12—H12	119.4
C4—C3—C2	120.81 (14)	C12—C13—C8	119.28 (14)
C3—C4—C5	118.60 (14)	C12—C13—H13	120.4
C3—C4—H4	120.7	C8—C13—H13	120.4
C5—C4—H4	120.7	C11—C14—H14A	109.5
C4—C5—C6	120.77 (14)	C11—C14—H14B	109.5
C4—C5—H5	119.6	H14A—C14—H14B	109.5
C6—C5—H5	119.6	C11—C14—H14C	109.5
C5—C6—C1	119.61 (14)	H14A—C14—H14C	109.5
C5—C6—C7	118.97 (13)	H14B—C14—H14C	109.5
C1—C6—C7	121.40 (13)		
			
C7—N1—N2—S1	172.11 (11)	C5—C6—C7—N1	−165.16 (14)
O1—S1—N2—N1	49.24 (12)	C1—C6—C7—N1	13.7 (2)
O2—S1—N2—N1	177.34 (10)	O1—S1—C8—C13	−12.85 (14)
C8—S1—N2—N1	−67.26 (12)	O2—S1—C8—C13	−144.84 (13)
C6—C1—C2—F1	−177.38 (13)	N2—S1—C8—C13	103.15 (13)
C6—C1—C2—C3	1.0 (2)	O1—S1—C8—C9	165.65 (11)
F1—C2—C3—F2	−1.6 (2)	O2—S1—C8—C9	33.66 (13)
C1—C2—C3—F2	179.95 (13)	N2—S1—C8—C9	−78.36 (13)
F1—C2—C3—C4	177.91 (14)	C13—C8—C9—C10	0.6 (2)
C1—C2—C3—C4	−0.5 (2)	S1—C8—C9—C10	−177.85 (12)
F2—C3—C4—C5	178.96 (13)	C8—C9—C10—C11	0.3 (2)
C2—C3—C4—C5	−0.5 (2)	C9—C10—C11—C12	−1.0 (3)
C3—C4—C5—C6	1.1 (2)	C9—C10—C11—C14	179.77 (16)
C4—C5—C6—C1	−0.6 (2)	C10—C11—C12—C13	0.7 (3)
C4—C5—C6—C7	178.21 (13)	C14—C11—C12—C13	179.94 (17)
C2—C1—C6—C5	−0.4 (2)	C11—C12—C13—C8	0.2 (2)
C2—C1—C6—C7	−179.24 (13)	C9—C8—C13—C12	−0.9 (2)
N2—N1—C7—C6	175.73 (11)	S1—C8—C13—C12	177.54 (12)

### Hydrogen-bond geometry (Å, º)

**Table:**

*D*—H···*A*	*D*—H	H···*A*	*D*···*A*	*D*—H···*A*
N2—H2···O1^i^	0.89 (1)	2.09 (1)	2.9747 (18)	174 (2)
C1—H1···F1^ii^	0.95	2.47	3.345 (2)	153
C4—H4···O2^iii^	0.95	2.57	3.473 (2)	159

Symmetry codes: (i) *x*+1, *y*, *z*; (ii) −*x*, −*y*+1, −*z*+1; (iii) *x*+1, −*y*+3/2, *z*+1/2.
