# Supplementary figures and images for: Crystal structure of (E)-N′-(3,4-di­fluoro­benzyl­idene)-4-methyl­benzene­sulfono­hydrazide
Source: Acta Crystallogr E Crystallogr Commun. 2015 Sep 17;71(Pt 10):o761. doi: 10.1107/S2056989015016205 (PMC4647373; doi:10.1107/S2056989015016205)

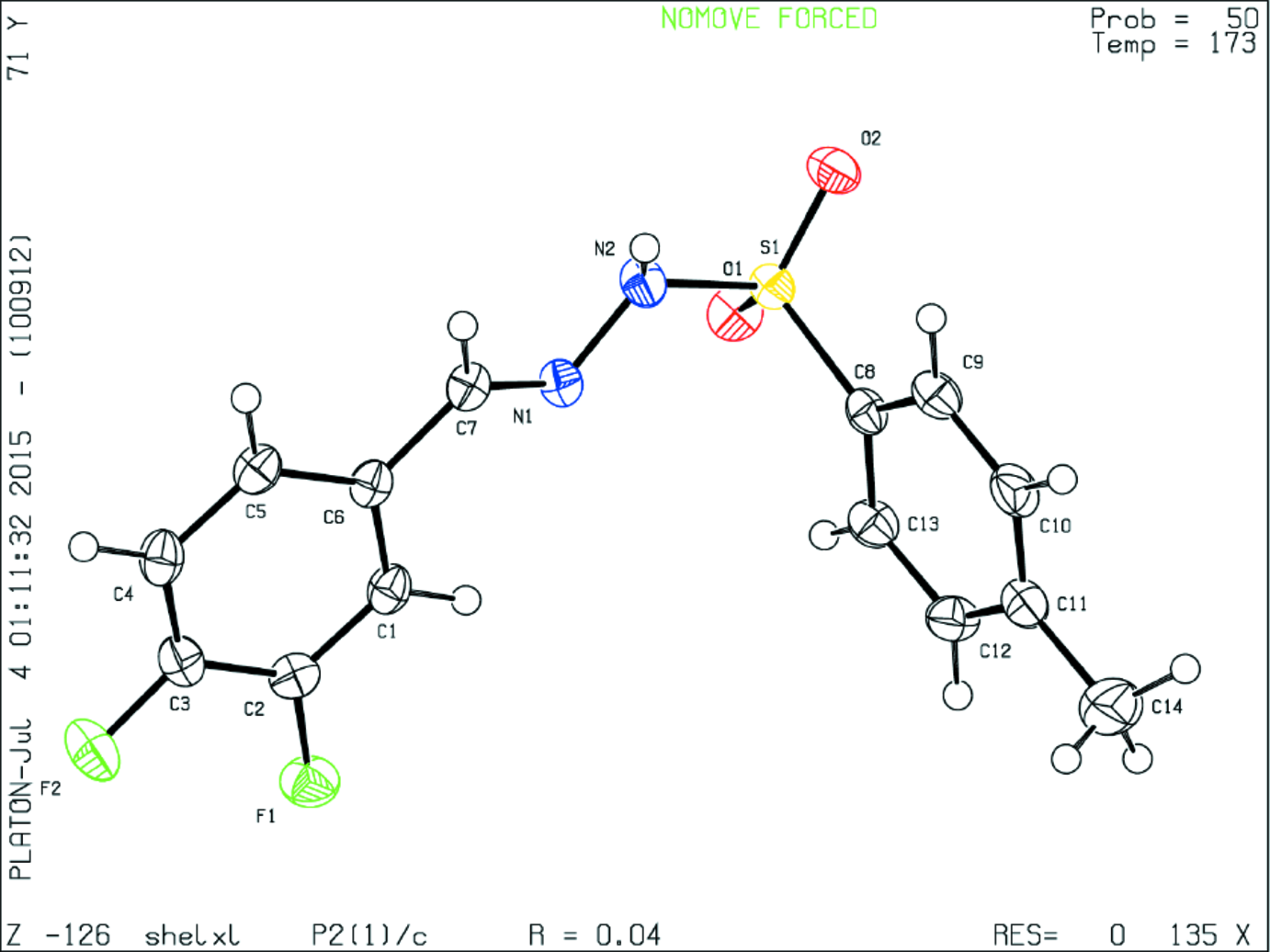

Supplement: Supplementary file 4 [file e-71-0o761-fig1.tif]
